# Matrix quality and disturbance frequency drive evolution of species behavior at habitat boundaries

**DOI:** 10.1002/ece3.1841

**Published:** 2015-11-24

**Authors:** Amanda E. Martin, Lenore Fahrig

**Affiliations:** ^1^ Geomatics and Landscape Ecology Laboratory (GLEL)Ottawa‐Carleton Institute of BiologyCarleton University209 Nesbitt Biology Building1125 Colonel By DriveOttawaOntarioK1S 5B6Canada

**Keywords:** Boundary avoidance, edge avoidance, emigration, habitat border, landscape context, natural selection

## Abstract

Previous theoretical studies suggest that a species' landscape should influence the evolution of its dispersal characteristics, because landscape structure affects the costs and benefits of dispersal. However, these studies have not considered the evolution of boundary crossing, that is, the tendency of animals to cross from habitat to nonhabitat (“matrix”). It is important to understand this dispersal behavior, because of its effects on the probability of population persistence. Boundary‐crossing behavior drives the rate of interaction with matrix, and thus, it influences the rate of movement among populations and the risk of dispersal mortality. We used an individual‐based, spatially explicit model to simulate the evolution of boundary crossing in response to landscape structure. Our simulations predict higher evolved probabilities of boundary crossing in landscapes with more habitat, less fragmented habitat, higher‐quality matrix, and more frequent disturbances (i.e., fewer generations between local population extinction events). Unexpectedly, our simulations also suggest that matrix quality and disturbance frequency have much stronger effects on the evolution of boundary crossing than either habitat amount or habitat fragmentation. Our results suggest that boundary‐crossing responses are most affected by the costs of dispersal through matrix and the benefits of escaping local extinction events. Evolution of optimal behavior at habitat boundaries in response to the landscape may have implications for species in human‐altered landscapes, because this behavior may become suboptimal if the landscape changes faster than the species' evolutionary response to that change. Understanding how matrix quality and habitat disturbance drive evolution of behavior at boundaries, and how this in turn influences the extinction risk of species in human‐altered landscapes should help us identify species of conservation concern and target them for management.

## Introduction

Dispersal among habitat patches has both costs and benefits, and the effects of these on fitness should drive evolution of dispersal characteristics that minimize the cost: benefit ratio. The primary cost is the risk of mortality in the “matrix”, that is, the nonhabitat parts of the landscape (Bonte et al. 2012). And, even if the individual survives, the energy expended during dispersal may compromise its fitness (Baker and Rao 2004; Bonte et al. 2012). The primary benefits of dispersal are that it allows individuals to track available resources and escape declining local conditions (Tellería and Pérez‐Tris 2003). Dispersal also allows individuals to avoid competition, inbreeding, and predation (Bollinger et al. 1993; Cronin et al. 2004; Moore et al. 2006).

The attributes of a species' landscape should influence the evolution of dispersal characteristics. For example, in landscapes with less habitat, the costs of dispersal should be higher because individuals will spend more time in the matrix (Baker and Rao 2004; Johnson et al. 2009). Similarly, individuals will spend more time in the matrix in landscapes with more fragmented habitat, where habitat fragmentation refers to the level of patchiness of habitat, for a given habitat amount. In contrast, in landscapes where disturbances are frequent, optimal dispersal rates should be high because dispersal allows individuals to escape declining local conditions (Friedenberg 2003). Thus, differences in dispersal characteristics among species and populations are likely at least partly explained by differences in their landscapes (Baguette et al. 2000; Merckx et al. 2003; Schtickzelle et al. 2006).

Previous studies support this idea. For example, models predict higher dispersal rates in landscapes with more habitat, less fragmented habitat, more dynamic habitat, and higher‐quality matrix (Travis and Dytham 1999; Heino and Hanski 2001; Poethke and Hovestadt 2002; Bonte and De La Peña 2009; Poethke et al. 2011). In addition, movement pathways are predicted to be straighter in landscapes with less habitat, less fragmented habitat, and lower‐quality matrix (Zollner and Lima 1999; Bartoń et al. 2009; Travis et al. 2012).

Previous theoretical studies of the evolution of dispersal characteristics have not considered evolution of the tendency to cross habitat boundaries when they are encountered. When a dispersing individual encounters a habitat boundary, does it turn back into habitat, or cross into the matrix? Responses to habitat–matrix boundaries have been observed in insects, amphibians, mammals, and birds (Basquill and Bondrup‐Nielsen 1999; Ries and Debinski 2001; Rodríguez et al. 2001; Merckx et al. 2003; Schtickzelle and Baguette 2003; Rittenhouse and Semlitsch 2006). The boundary‐crossing response is important for population persistence, particularly in a human‐altered landscape, because it drives rates of interaction with human‐dominated areas of the landscape.

Although empirical studies have compared rates of boundary crossing between populations in different landscapes (e.g., Baguette et al. 2003; Merckx et al. 2003; Schtickzelle and Baguette 2003; Schtickzelle et al. 2006), these studies do not tell us how the different attributes of these landscapes affect the evolution of behavior at habitat boundaries. This is because the landscape attributes were intercorrelated. For example, Schtickzelle and Baguette (2003) found boundary crossing was less likely in landscapes with abundant, unfragmented habitat and high‐quality matrix than landscapes with rare, fragmented habitat and low‐quality matrix, but we do not know which of these landscape attributes drove the boundary‐crossing response. Understanding how different landscape attributes affect the evolution of boundary crossing should help us understand how to change the pattern of human landscape change to reduce its negative impacts on wildlife. For example, if we want to promote dispersal among habitat fragments, to allow for recolonization and rescue of small populations, we could focus on maintaining landscape attributes that favor high rates of boundary crossing.

We predict that the optimal probability of boundary crossing should be higher in landscapes with more habitat that is less fragmented. This is because species in landscapes with more habitat and less fragmented habitat should encounter matrix less often. In this case, individuals will rarely experience the cost of dispersal through matrix, resulting in weaker selection for avoidance of boundary crossing. Additionally, the time spent in the matrix should be lower and the chance of finding new habitat should be higher than in landscapes with less habitat that is more fragmented. In addition, boundary crossing should be higher when matrix quality is higher, as the risk of movement into the matrix is reduced. We also predict that the optimal probability of boundary crossing should be higher in landscapes that are more dynamic, for example, where disturbances are more frequent, as the benefit of dispersal is higher in such landscapes.

Here, we evaluate these predictions by simulating the evolution of the boundary‐crossing response in landscapes that differ in habitat amount, habitat fragmentation, matrix quality, and disturbance frequency.

## Materials and Methods

### Overview

Our modeling framework was based on previously published individual‐based, spatially explicit models of the evolution of dispersal in response to landscape structure (Travis and Dytham 1998, 1999). We simulated population dynamics and the evolution of the boundary‐crossing response in landscapes that varied in habitat amount, habitat fragmentation, matrix quality, and disturbance frequency. Evolution of the boundary‐crossing response occurred because the probability of an individual crossing from habitat to matrix when it encountered a habitat boundary, that is, its probability of boundary crossing, was simulated as a heritable trait. We measured the boundary‐crossing response as both the evolved population mean of the boundary‐crossing trait value, and the actual per capita rate of boundary crossing during the simulation, that is, the proportion of the population that crossed from habitat to matrix. We included the actual per capita rate of boundary crossing because it reflects the interacting effects of the evolved boundary‐crossing trait and the landscape; how frequently an individual with a given probability of boundary crossing actually crosses a habitat–matrix boundary depends on how frequently it encounters boundaries during dispersal. To evaluate our predictions, we related each of the two measures of the boundary‐crossing response to habitat amount, fragmentation, matrix quality, and disturbance frequency.

Because dispersal characteristics coevolve in response to landscape structure, in addition to the boundary‐crossing response, we included evolution of three other dispersal characteristics as independent, heritable traits: (1) dispersal propensity, or the probability that an individual disperses; (2) path straightness in matrix; and (3) path straightness in habitat. We interpreted effects of these additional characteristics on the evolution of boundary crossing as indirect effects of the landscape structure on the evolution of boundary crossing. For example, if evolved dispersal paths are straighter in certain landscapes, and path straightness influences the evolved boundary‐crossing response, then the optimal boundary‐crossing response is indirectly affected by landscape structure.

### Model description

**Figure 1:**
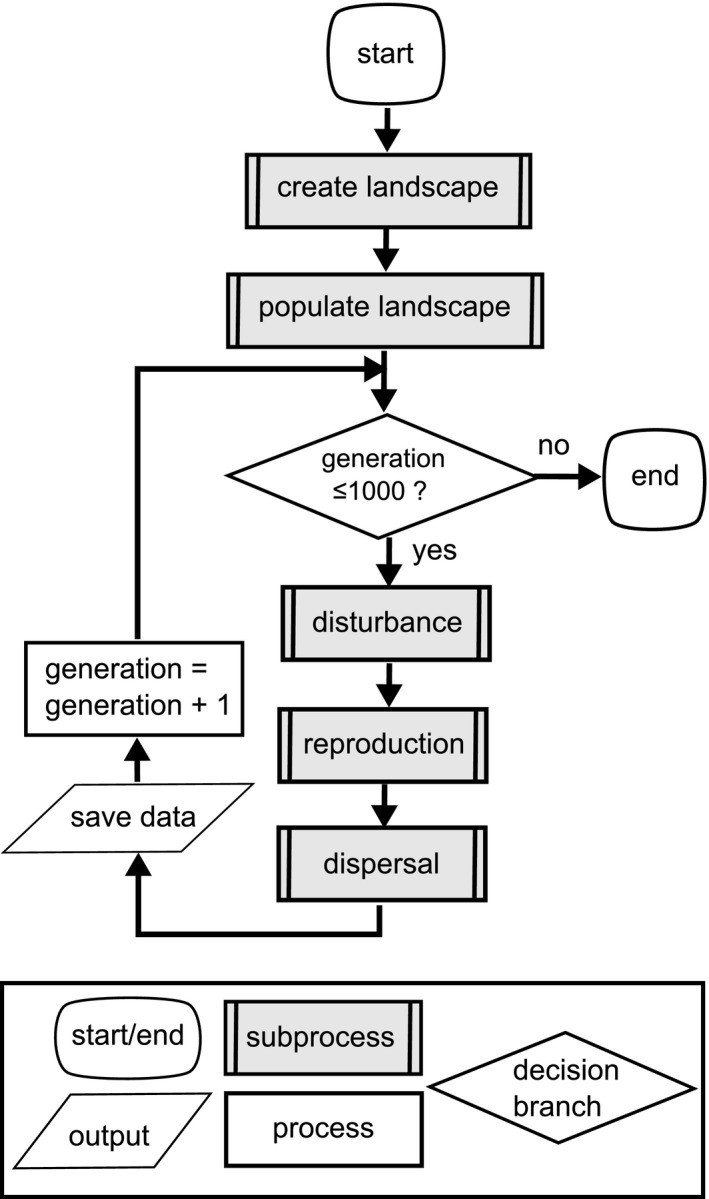
Flow chart of the simulation model. See Appendix S1 for flow charts for each of the five subprocesses.

We constructed the model in NetLogo (Wilensky 1999). Each generation involved (1) habitat disturbance, resulting in some local population extinctions; (2) density‐dependent reproduction, including transfer of genetic information; followed by (3) dispersal, that is, movement of individuals from their birth place, resulting in either dispersal mortality or settlement in a new location (Fig. 1). See Appendix S1 for additional flow diagrams and Appendix S2 for model parameters.

### Create landscape

**Figure 2:**
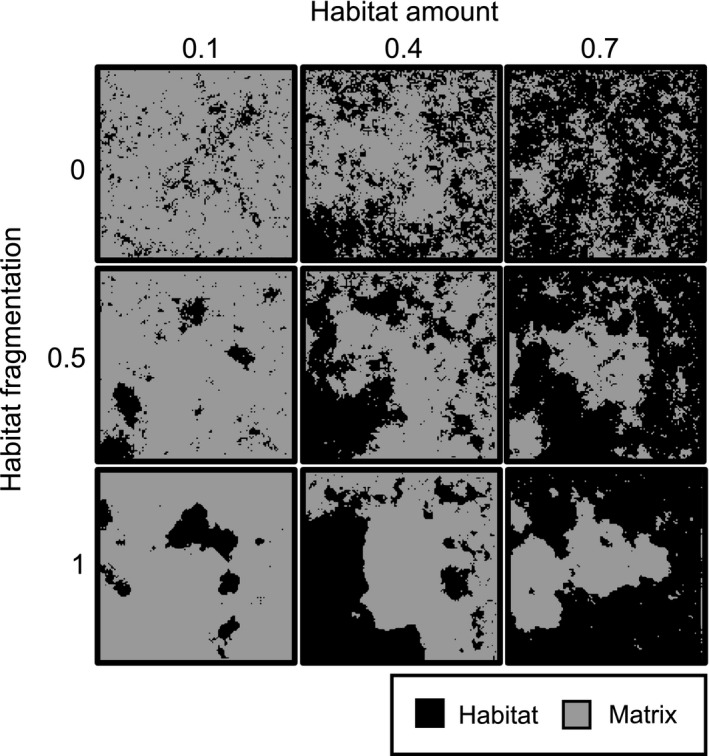
Examples of the artificial landscapes created through the midpoint displacement algorithm (Saupe 1988). Habitat amount was the proportion of the landscape in habitat. Habitat fragmentation was determined by the Hurst exponent, which controls the autocorrelation in a fractal surface created through the midpoint displacement algorithm, and sets the level of patchiness for a given habitat amount. We simulated population dynamics and the evolution of the boundary‐crossing response in 1000 different landscapes, with habitat amounts ranging from 0.1 to 0.7, and habitat fragmentation ranging from 0 to 1.

Each simulation began by creating a square 127 × 127 (16,129) grid of cells, with each cell assigned as habitat or matrix. The differences between habitat and matrix were that reproduction could only occur in habitat cells, and dispersal mortality was lower in habitat than in matrix. To determine which cells were habitat and which were matrix, we used a midpoint displacement algorithm to generate a fractal surface (Saupe 1988). Fragmentation, independent of habitat amount, was controlled by the Hurst exponent (H), which determines the autocorrelation in a fractal surface. We superimposed the fractal surface on the landscape, and assigned the required proportion of cells (based on habitat amount) with the highest fractal values as habitat; remaining cells were matrix (Fig. 2). Matrix quality was assigned as the probability of dispersal mortality in matrix cells. We then identified habitat patches (for the disturbance algorithm; see below) as groups of contiguous habitat cells, based on a Moore neighborhood rule.

### Populate landscape

For each simulation run, we seeded the landscape with one individual per habitat cell. Each individual was assigned a random value for its initial probability of boundary crossing, dispersal propensity, path straightness in matrix, and path straightness in habitat.

### Habitat disturbance

Disturbance caused death of all individuals in a habitat patch. To spread disturbances over time, at the beginning of a run, we assigned each patch a number of generations until disturbance. This disturbance interval was randomly drawn from a Poisson distribution, with mean equal to the disturbance frequency. When a habitat patch reached its disturbance interval it was disturbed, after which a new disturbance interval was selected from the Poisson distribution.

### Reproduction and genetic transfer

We modeled an asexual, haploid species with nonoverlapping generations. The number of offspring produced by each adult in a habitat cell was randomly drawn from a Poisson distribution, where the mean for cell *i* in generation *t* was as follows:

(1) $$\mu_{i,t}=\lambda /\left(1+a\times N_{i,t}\right)$$

where *a* = (*λ* − 1)/*k*,* λ *= intrinsic growth rate, *k* = cell carrying capacity, and *N*_*i,t*_ = number of adults in cell *i* at generation *t* (Hassell 1975). This density‐dependent reproduction introduces within‐cell competition.

Offspring inherited the parental genotype for the four dispersal characteristics, subject to possible mutation of the gene controlling each. Mutation randomly increased or decreased the value of the dispersal characteristic by 0.01.

### Dispersal

Each juvenile dispersed or not, depending on its genetically determined dispersal propensity. A dispersing individual kept moving until it either found a new habitat cell, or died. Dispersal could be within or between habitat patches and was modeled as a series of movement steps of one cell‐length each. The change in direction between consecutive steps was randomly drawn from a wrapped Cauchy distribution with a mean of zero and a concentration parameter (*ρ*) which varied from 0 (uncorrelated) to 1 (straight line). The *ρ* was genetically determined for each individual, with different values for habitat and matrix. If a movement step would result in the individual crossing from habitat to matrix, its decision to cross or not depended on its genetically determined boundary‐crossing response. If the individual decided not to cross, it would either move in a randomly selected direction within the habitat or, if no such option existed, it remained in its current location. If a movement step would take an individual outside the landscape, a new direction was randomly selected such that it would remain within the landscape. Dispersal mortality was applied after each movement step. If the individual moved between a habitat cell and a matrix cell, the probability of mortality was the average of the two. After each movement step, if the individual was in a habitat cell with fewer than *k* individuals, it settled, otherwise it took another movement step.

### Testing the hypotheses

We simulated population dynamics and evolution of dispersal characteristics in 1000 different landscapes. We measured the evolved boundary‐crossing behavior after 1000 generations in two ways: (1) the evolved population mean boundary‐crossing trait; and (2) the actual per capita rate of boundary crossing.

To evaluate our predictions for the effects of landscape structure on the evolved boundary‐crossing response, we related each of these two measures to each landscape attribute: habitat amount, fragmentation, matrix quality, and disturbance frequency, using multiple linear regression in R (R Core Team 2014). We included quadratic terms for each predictor, to account for nonlinear relationships. We used the percent sum of squares (%SS) from an analysis of variance as a measure of variation explained by each landscape attribute, measured as

(2) $$\%\text{SS}=100\times {\text{SS}}_{p}/{\text{SS}}_{t}$$

where SS_*p*_ = sum of squared variation explained by a given attribute, and SS_*t*_ = total sum of squared variation around the grand mean (Jackson and Fahrig 2012).

Our predictions for the effects of habitat amount and fragmentation on the evolution of boundary crossing were, in part, based on the assumption that less frequent interaction with matrix results in weaker selection for avoidance of boundary crossing. If true, we expect to see greater within‐population variability in the evolved boundary‐crossing trait in landscapes where encounters with matrix are infrequent (i.e., in landscapes with abundant, unfragmented habitat). To test this, we measured the variance in the population boundary‐crossing trait and modeled the landscape effects on this measure of within‐population variability, as described above (Appendix S3).

To assess how our model compared to previously published simulation models (see Introduction), we also modeled the landscape effects on the remaining three dispersal characteristics: (1) dispersal propensity; (2) path straightness in matrix; and (3) path straightness in habitat after the 1000th generation, as described above (Appendix S4).

## Results

**Figure 3:**
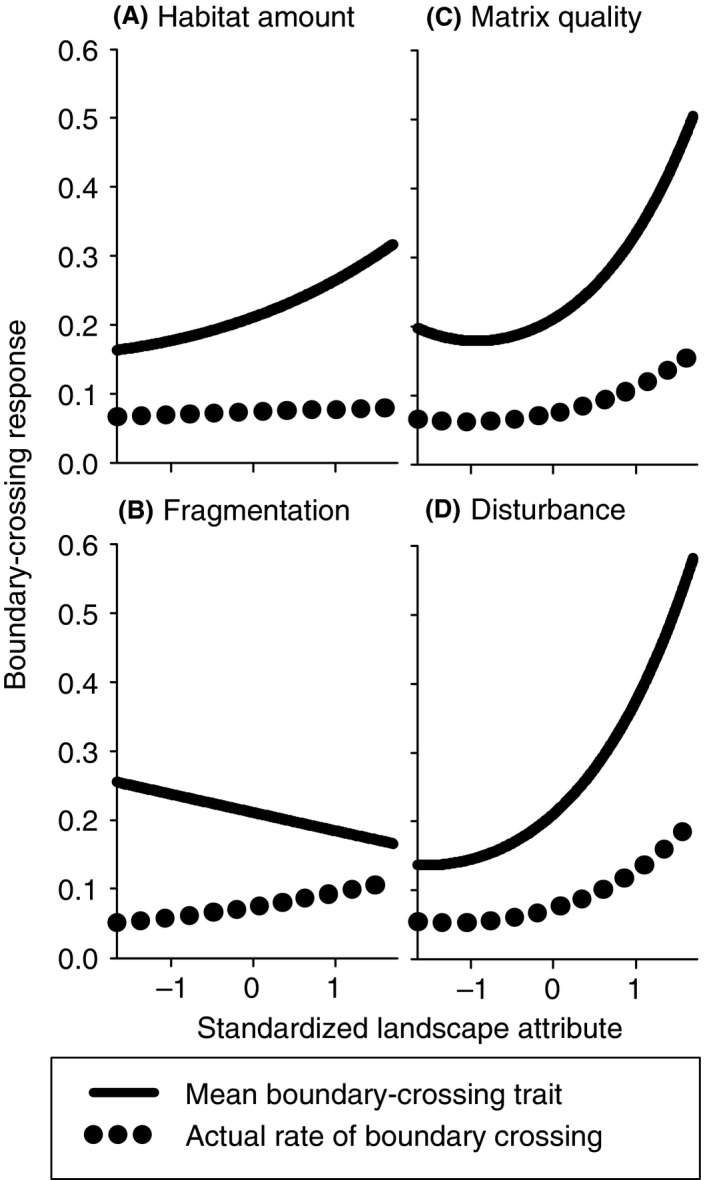
Effects of (A) habitat amount, (B) habitat fragmentation, (C) matrix quality, and (D) disturbance frequency on each of the two measures of the evolved boundary‐crossing response, when holding all other landscape attributes at their mean values. The evolved boundary‐crossing response was measured in two ways, as the population mean boundary‐crossing trait, and the actual per capita rate of boundary crossing. Standardized landscape attribute values were scaled such that larger values indicate more habitat, more fragmented habitat, higher matrix quality, and more frequent disturbance. Relationships were modeled by multiple linear regression, using square‐root‐transformed population mean boundary‐crossing traits and square‐root‐transformed per capita rates of boundary crossing (back‐transformed prior to plotting), for the 1000 simulation runs. We included quadratic terms for each predictor, to account for nonlinear relationships.

The simulation results supported our predictions for the effects of landscape structure on the evolved boundary‐crossing trait. The mean probability of boundary crossing increased in landscapes with more habitat and less fragmented habitat (Fig. 3A and B). Species evolved higher probabilities of boundary crossing in landscapes with higher‐quality matrix and more frequent habitat disturbance (Fig. 3C and D). The evolution of boundary crossing was largely driven by matrix quality and habitat disturbance; the %SS for matrix quality was more than three times the %SS for either habitat amount or fragmentation, and the %SS for disturbance frequency was more than six times the %SS for either habitat amount or fragmentation (Table 1).

**Table 1:** Percent sum of squares (%SS), for a multiple linear regression model of the relationship between each of the two measures of the boundary‐crossing response (i.e., the evolved population mean boundary‐crossing trait and the actual per capita rate of boundary crossing, after 1000 generations) and the four landscape attributes. We included quadratic terms for each predictor, to account for nonlinear relationships. %SS combines the variance explained by both the linear and quadratic terms

Attribute	Boundary‐crossing trait	Rate of boundary crossing
Habitat amount	6.80	0.56
Habitat fragmentation	2.62	10.72
Matrix quality	22.66	21.18
Disturbance frequency	42.03	41.65
Residual	25.89	25.89

The relationships between the actual per capita rate of boundary crossing and landscape structure generally mirrored the relationships between the evolved boundary‐crossing trait and landscape structure discussed above (Fig. 3). Matrix quality and disturbance frequency explained more of the variation in the rate of boundary crossing after 1000 generations than either habitat amount or fragmentation (Table 1), with increasing rates of boundary crossing in landscapes with higher matrix quality and more frequent disturbance. The one exception was that the evolved probability of boundary crossing decreased with habitat fragmentation, while the actual per capita rate of boundary crossing increased with habitat fragmentation (Fig. 3B).

## Discussion

Our simulation results support the hypothesis that boundary‐crossing behavior evolves in response to landscape structure. To our knowledge, this is the first theoretical study to examine the independent effects of different landscape attributes on the evolution of behavior at habitat boundaries. The boundary‐crossing response is important for population persistence, because it influences the rate of movement among habitat patches (Brown and Kodric‐Brown 1977; Hanski et al. 1995). It may be particularly important in human‐altered landscapes, where populations may only persist if individuals can move among habitat remnants within a human‐dominated matrix.

Species evolved to cross boundaries more readily in landscapes with more frequent disturbance. More frequent disturbance increases the benefits of dispersal relative to its costs, because boundary crossing allows individuals to escape declining local conditions and access unexploited habitat. While previously hypothesized (Fahrig 2007), the effect of disturbance on evolution of behavior at boundaries has not, to our knowledge, been studied before.

Species evolved to avoid crossing from habitat to matrix in landscapes with lower‐quality matrix, allowing individuals to avoid the higher cost of dispersal in a lower‐quality matrix. The effect of matrix quality on the evolution of behavior at boundaries has not been independently studied before. However, the relationship we found is consistent with studies finding fewer boundary crossings into lower‐quality matrix than into higher‐quality matrix (Haynes and Cronin 2003; Stevens et al. 2006).

Species evolved higher probabilities of boundary crossing in landscapes with more habitat and less fragmented habitat, as observed in empirical studies (Merckx et al. 2003; Schtickzelle and Baguette 2003). This appears to be because there is a higher chance of successful dispersal through matrix when habitat patches are larger and less time is spent in matrix. We also expected evolution of higher mean probabilities of boundary crossing in landscapes with more habitat and less fragmented habitat because individuals in these landscapes rarely experience the cost of dispersal through matrix, resulting in weaker selection for avoidance of boundary crossing; however, we found little evidence of weaker selection in landscapes with more abundant, less fragmented habitat (Appendix S3).

Although landscape structure generally had the same effects on the evolved boundary‐crossing trait and the actual per capita rates of boundary crossing, we did find one exception: the evolved probability of boundary crossing decreased with habitat fragmentation, while the per capita rate of boundary crossing increased with habitat fragmentation. This is because the actual per capita rate of boundary crossing results from the combined effects of the evolved boundary‐crossing trait and the frequency of encounters with boundaries. For a given probability of boundary crossing, there should be more frequent boundary crossings when habitat patches are smaller (Baguette et al. 2003; Schtickzelle and Baguette 2003; Schtickzelle et al. 2006). Thus, individuals in landscapes with more fragmented habitat encountered habitat boundaries more frequently than individuals in landscapes with less fragmented habitat, resulting in more actual boundary‐crossing events in fragmented landscapes, even though the probability of crossing per boundary interaction was lower.

Surprisingly, our simulations suggest that habitat amount and habitat fragmentation have weaker effects on the evolution of boundary crossing than matrix quality or disturbance frequency. This suggests that boundary‐crossing responses are most affected by the cost of dispersal through the matrix, and the benefit of escaping local extinction events. Based on this result, we recommend that researchers focus on the roles of matrix quality and disturbance, because these have potentially larger effects on the costs and benefits of boundary crossing than either habitat amount or fragmentation. It also suggests we should be cautious in attributing differences in evolved boundary‐crossing responses between landscapes to habitat amount or fragmentation when these landscape attributes are correlated with either matrix quality or disturbance. For example, differences in the evolved boundary‐crossing behavior of speckled wood butterflies (*Pararge aegeria*) between a woodland landscape and a high‐intensity agricultural landscape may be driven by differences in matrix quality between these two landscapes, rather than differences in the availability of forested areas (Merckx et al. 2003).

### Model evaluation

Our model extensions, to include behavior at habitat boundaries, did not alter previous theoretical findings on the evolution of other dispersal characteristics. In particular, our predictions for evolution of dispersal propensities were consistent with previous studies: higher dispersal propensities in landscapes with more habitat, less fragmented habitat, higher‐quality matrix, and more frequent disturbance (Appendix S4; Travis and Dytham 1999; Heino and Hanski 2001; Poethke and Hovestadt 2002; Bonte and De La Peña 2009; Poethke et al. 2011). However, landscape effects on the evolution of the dispersal propensity were weaker than expected from previous studies, likely because evolution of boundary‐crossing behavior offset the costs of dispersal, reducing the landscape effects on the dispersal propensity. Also consistent with previous studies, we found selection for straighter dispersal paths in matrix when there was less habitat, less fragmented habitat, and lower‐quality matrix (Appendix S4; Zollner and Lima 1999; Bartoń et al. 2009; Travis et al. 2012).

We did not model the context‐dependent conditions that may affect an individual's behavior at habitat boundaries. For example, we did not allow for a density‐dependent boundary‐crossing response, with individuals more willing to cross habitat–matrix boundaries when local densities are high (Enfjäll and Leimar 2005). We also did not include effects of body condition on the boundary‐crossing response, although previous studies suggest an individual is more likely to disperse when its body condition is good (Meylan et al. 2002; Barbraud et al. 2003). We speculate that context‐dependent boundary‐crossing behaviors would increase the within‐population variability in responses to boundaries relative to what we modeled and thus may reduce the strength of relationship between genetic determinants of boundary‐crossing and landscape structure. However, we do not expect them to affect the direction of the relationships between the boundary‐crossing response and landscape attributes, because context‐dependent behaviors do not alter how a given landscape attribute affects the overall costs and benefits of boundary crossing.

## Conclusions

Overall, our simulations suggest that landscape structure influences evolution of behavior at habitat boundaries. To date, empirical studies of behavior at habitat boundaries have shown that human landscape change affects species behavior at habitat boundaries (e.g., Baguette et al. 2003; Merckx et al. 2003; Schtickzelle and Baguette 2003; Schtickzelle et al. 2006). Future research should focus on how different landscape attributes affect the species' behavior at habitat boundaries. In particular, our simulations suggest that future research should focus on the roles of matrix quality and disturbance in the evolution of this dispersal characteristic.

Although landscape attributes are typically correlated in real landscapes, there are ways to minimize correlations among landscape attributes in empirical studies. First, correlations among landscape attributes can be minimized during site selection, by defining ranges of “low” and “high” values for each attribute and randomly selecting an equal number of study landscapes from all combinations of the low and high ranges of the attributes (Appendix S5; Pasher et al. 2013). We also recommend using standardized partial regression coefficients from multiple regression to indicate relative importance of landscape attributes, as these provide unbiased estimates of relative importance, even when predictors are correlated (Smith et al. 2009).

Additionally, our simulations suggest that relationships between boundary‐crossing behavior and landscape attributes can depend on whether one measures an intrinsic propensity to cross boundaries (i.e., the probability of boundary crossing per boundary interaction) or the actual rate of boundary crossing in the landscape context. This is because in some cases (e.g., with habitat fragmentation) these different measurements may lead to opposite conclusions about the relationship between boundary‐crossing behavior and landscape attributes. Therefore we suggest that future studies should include both measurements of boundary crossing.

Understanding how the landscape attributes drive evolution of a species' behavior at boundaries, and how this in turn influences the extinction risk of species in human‐altered landscapes, should help us identify species of conservation concern and manage landscapes for population persistence. Species with low probabilities of boundary crossing may be prone to extinction from habitat loss, because they are less able to recolonize after local extinctions or rescue small populations when habitat is lost. If true, we may want to manage for the landscape attributes that most strongly favor high rates of boundary crossing. For example, our simulations suggest that improving matrix quality in human‐altered landscapes should favor high rates of boundary crossing even when habitat is lost.

## Data accessibility

Simulation data set: uploaded as online supporting information. Simulation model: archived with the NetLogo User Community Models (http://ccl.northwestern.edu/netlogo/models/community/).

## Conflict of Interest

None declared.

## Supporting information


**Appendix S1**. Flow diagrams for each of the five simulation model subprocesses.

**Appendix S2**. Parameters used in the simulation model.

**Appendix S3**. Effects of landscape structure on the within‐population variability in the population boundary‐crossing trait.

**Appendix S4**. Effects of landscape structure on the evolution of dispersal propensity, path shape in matrix, and path shape in habitat.

**Appendix S5**. Example of site selection to minimize correlations between landscape attributes.

**Data S1**. Simulation data set.
