# Supplementary material for: Matrix quality and disturbance frequency drive evolution of species behavior at habitat boundaries
Source: Ecol Evol. 2015 Nov 24;5(24):5792–800. doi: 10.1002/ece3.1841 (PMC4717347; doi:10.1002/ece3.1841)

Appendix S1. Flow diagrams for each of the five simulation model subprocesses.


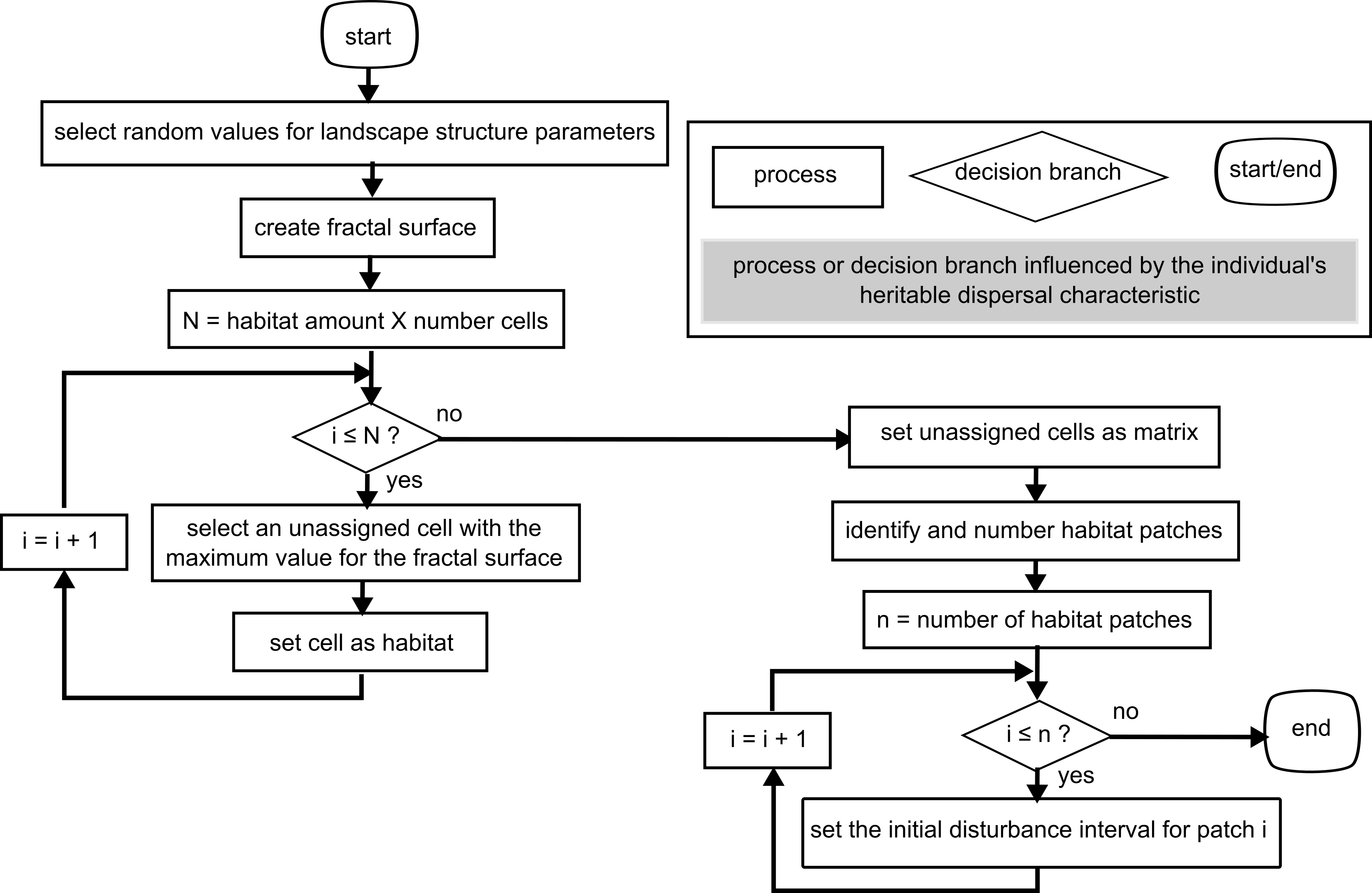


Fig. S1.1. Flow chart for the ‘create landscape’ subprocess of the simulation model (see Fig. 1 for the model overview).


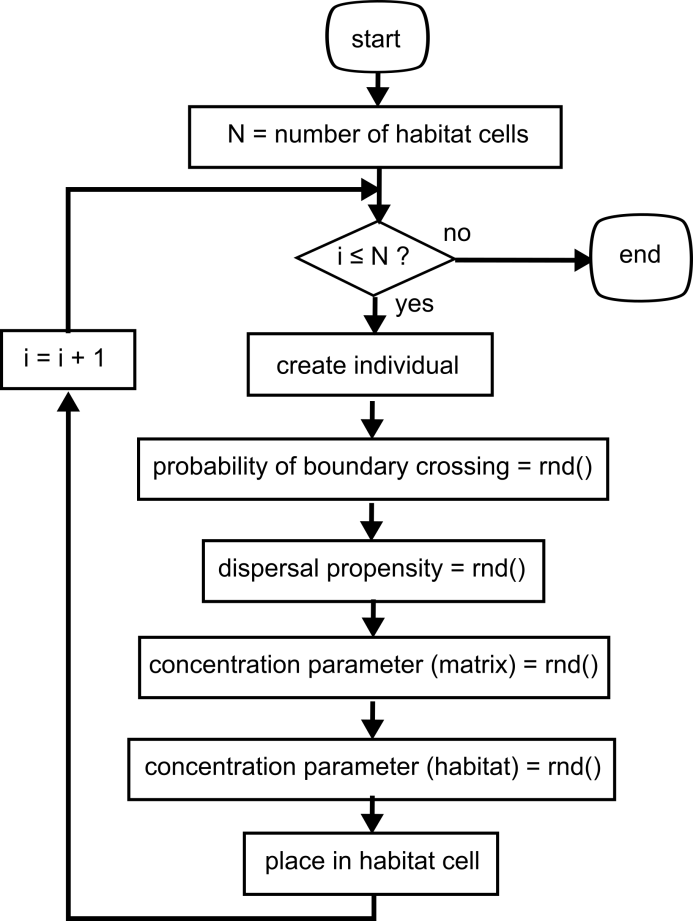


Fig. S1.2. Flow chart for the ‘populate landscape’ subprocess of the simulation model (see Fig. 1 for the model overview). See Fig. S1.1 for the flow chart legend. rnd() = random number between 0 and 1, drawn from a uniform distribution.


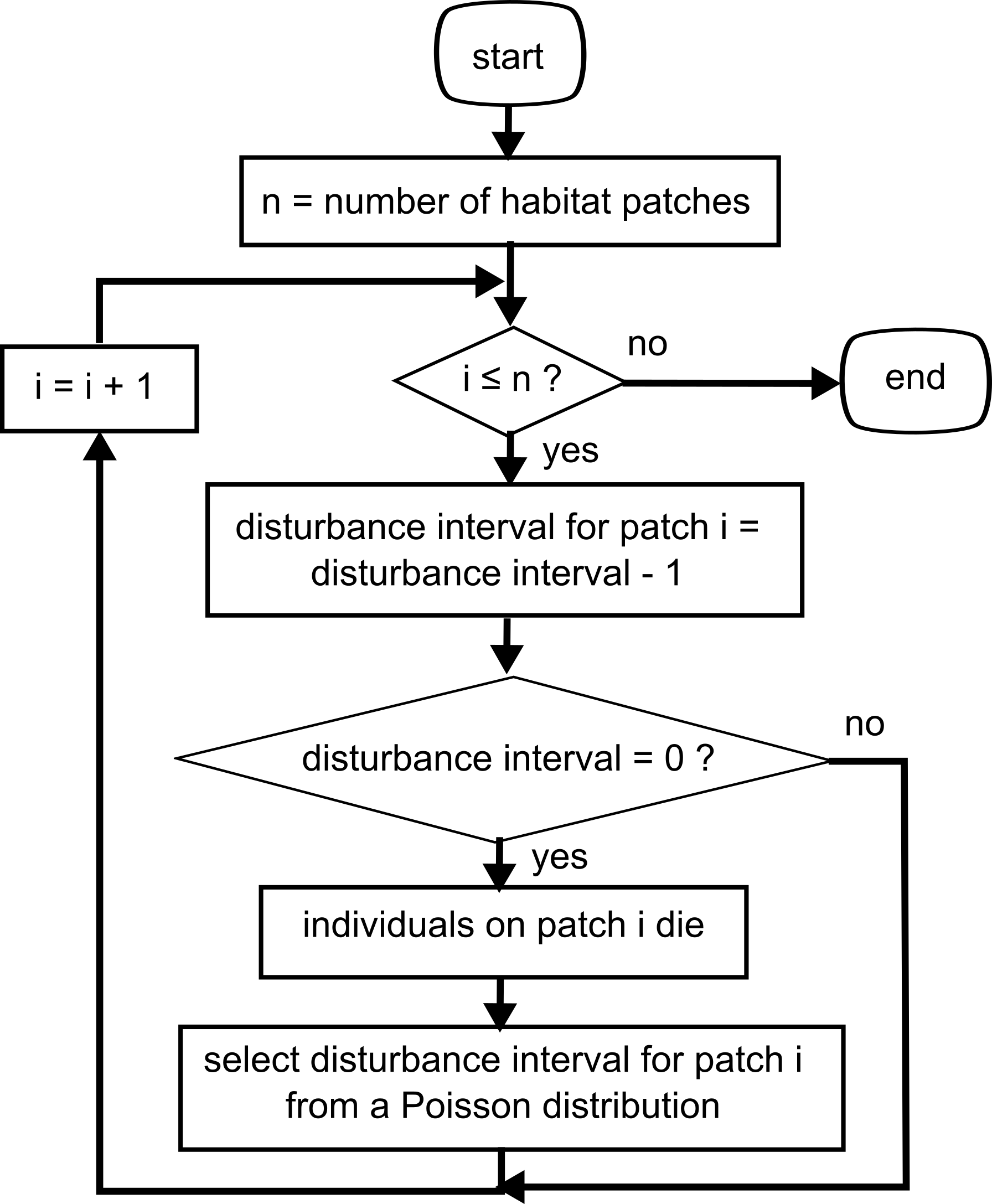


Fig. S1.3. Flow chart for the ‘disturbance’ subprocess of the simulation model (see Fig. 1 for the model overview). See Fig. S1.1 for the flow chart legend.


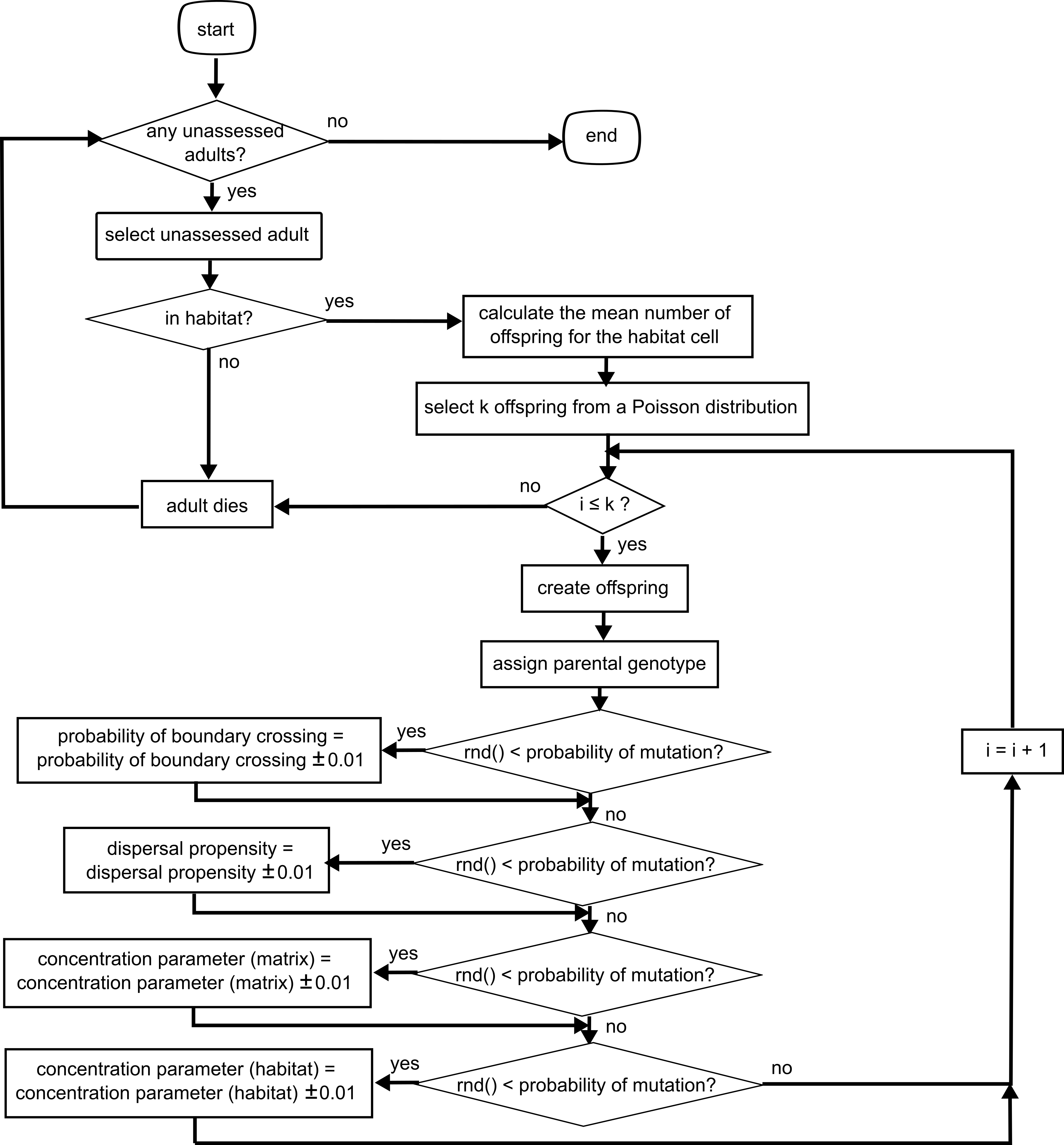


Fig. S1.4. Flow chart for the ‘reproduction’ subprocess of the simulation model (see Fig. 1 for the model overview). See Fig. S1.1 for the flow chart legend. rnd() = random number between 0 and 1, drawn from a uniform distribution.

Fig. S1.5. Flow chart for the ‘dispersal’ subprocess of the simulation model (see Fig. 1 for the model overview). See Fig. S1.1 for the flow chart legend. rnd() = random number between 0 and 1, drawn from a uniform distribution.


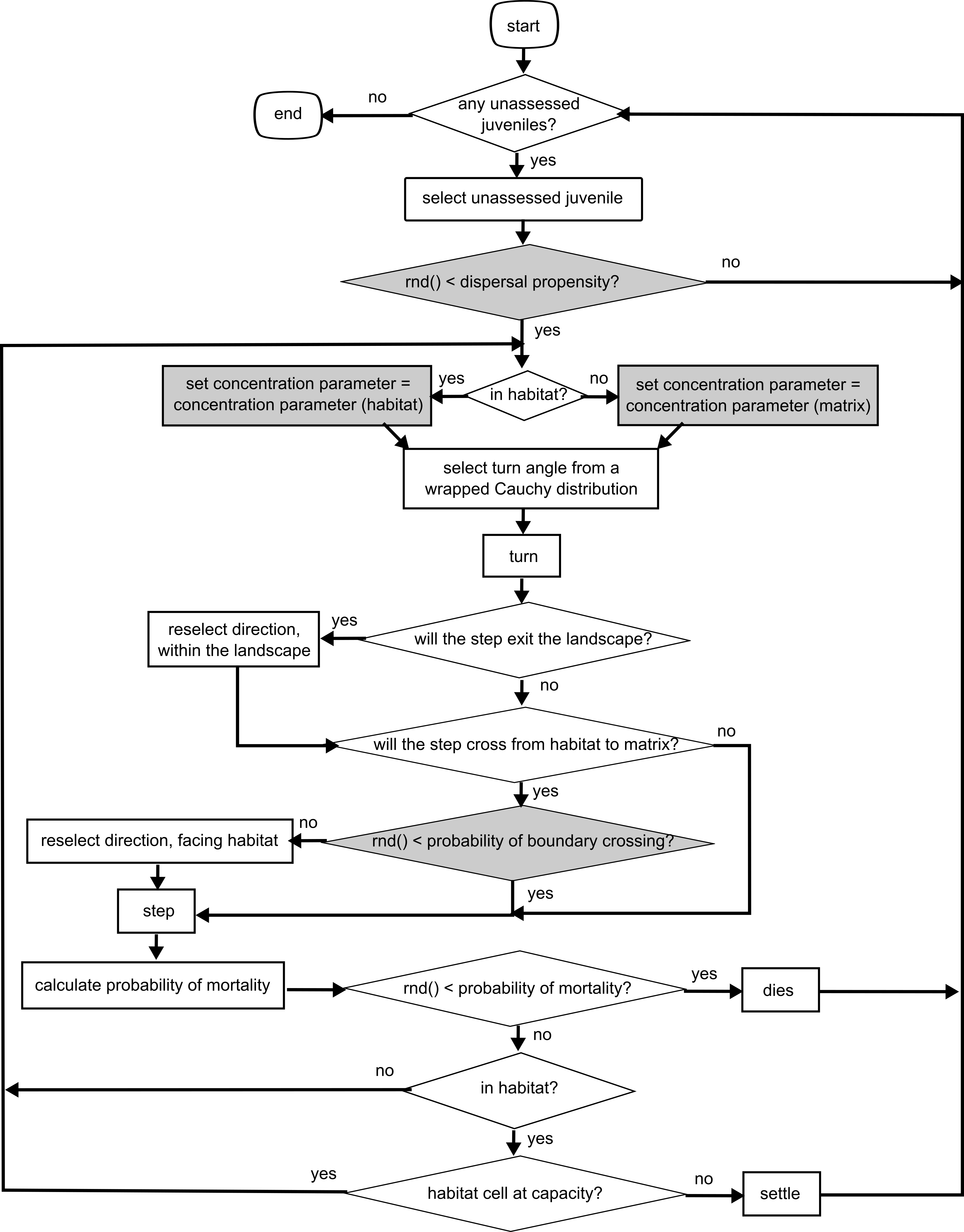

Supplement: Supplementary file 1 — Appendix S1. Flow diagrams for each of the five simulation model subprocesses. [file ECE3-5-5792-s001.docx]
