# Supplementary material for: Matrix quality and disturbance frequency drive evolution of species behavior at habitat boundaries
Source: Ecol Evol. 2015 Nov 24;5(24):5792–800. doi: 10.1002/ece3.1841 (PMC4717347; doi:10.1002/ece3.1841)
Supplement: Supplementary file 2 — Appendix S2. Parameters used in the simulation model. [file ECE3-5-5792-s002.docx]

Appendix S2. Parameters used in the simulation model.

Table S2. Parameters used in the simulation model. Subheadings indicate parameters that were kept constant for all simulation runs (constants), parameters that were varied between simulation runs but were constant within a given run (landscape attributes), and parameters that were allowed to vary among individuals and to evolve in response to the costs and benefits of dispersal (evolved dispersal characteristics).

| Parameter | Description | Value(s) |
| --- | --- | --- |
| **Constants** | | |
| landscape size | number of grid cells (dimensions) | 16,129 cells (127 × 127) |
| generations |  | 1000 |
| habitat quality | probability of mortality during dispersal across a habitat cell | 0.005 |
| intrinsic rate of increase (λ) | variable in the model of the mean number of offspring produced by each adult inhabiting a habitat cell (eqn 1) | 3 |
| cell carrying capacity (k) | threshold number of offspring supported by a habitat cell, involved in determining the mean number of offspring (eqn 1) and settlement during dispersal | 6 |
| mutation rate | probability of mutation, leading to a change in the value for the target dispersal characteristic (±0.01) | 0.005 |
| **Landscape attributes** | | |
| habitat amount | proportion of the grid cells in habitat; smaller values mean less habitat | 0.1 – 0.7 |
| habitat fragmentation | Hurst exponent, determining spatial autocorrelation of habitat; smaller values relate to greater fragmentation | 0 – 1 |
| matrix quality | probability of mortality during dispersal across a matrix cell; lower probabilities of mortality relate to higher quality | 0.006 – 0.2 |
| disturbance frequency | mean number of generations until disturbance, for a Poisson distribution; smaller values indicate more frequent disturbance | 10 – 100 |
| **Evolved dispersal characteristics** | | |
| dispersal propensity | probability of dispersal in a given generation | 0 – 1 |
| boundary crossing response | the probability of an individual crossing from habitat to matrix when it encountered a habitat boundary | 0 – 1 |
| path straightness in matrix | concentration parameter (ρ) for a wrapped Cauchy distribution; determines the degree of change in movement direction between successive movement steps originating in matrix | 0 – 1 |
| path straightness in habitat | concentration parameter (ρ) for a wrapped Cauchy distribution; determines the degree of change in movement direction between successive movement steps originating in habitat | 0 – 1 |
