# Supplementary material for: Matrix quality and disturbance frequency drive evolution of species behavior at habitat boundaries
Source: Ecol Evol. 2015 Nov 24;5(24):5792–800. doi: 10.1002/ece3.1841 (PMC4717347; doi:10.1002/ece3.1841)
Supplement: Supplementary file 3 — Appendix S3. Effects of landscape structure on the within‐population variability in the population boundary‐crossing trait. [file ECE3-5-5792-s003.docx]

Appendix S3. Effects of landscape structure on the within-population variability in the population boundary-crossing trait.

Table S3. Percent sum of squares (%SS), for a multiple linear regression model of the relationship between the ln-transformed variance in the population probability of boundary crossing after 1000 generations and the four landscape attributes, for 1000 simulation runs. We included quadratic terms for each predictor, to account for non-linear relationships. %SS combines the variance explained by both the linear and quadratic terms.

| Attribute | %SS |
| --- | --- |
| Habitat amount | 3.85 |
| Habitat fragmentation | 0.15 |
| Matrix quality | 2.23 |
| Disturbance frequency | 0.66 |
| Residual | 93.12 |





Fig. S3. Effects of (a) habitat amount, (b) habitat fragmentation, (c) matrix quality, and (d) disturbance frequency on the evolved variance in the population boundary-crossing trait, when holding all other landscape attributes at their mean values. Standardized landscape attribute values were scaled such that larger values indicate more habitat, more fragmented habitat, higher matrix quality, and more frequent disturbance. Relationships were modelled by multiple linear regression, using ln-transformed variance in the population boundary-crossing trait (back-transformed prior to plotting), for the 1000 simulation runs. We included quadratic terms for each predictor, to account for non-linear relationships.
