# Supplementary material for: Matrix quality and disturbance frequency drive evolution of species behavior at habitat boundaries
Source: Ecol Evol. 2015 Nov 24;5(24):5792–800. doi: 10.1002/ece3.1841 (PMC4717347; doi:10.1002/ece3.1841)
Supplement: Supplementary file 4 — Appendix S4. Effects of landscape structure on the evolution of dispersal propensity, path shape in matrix, and path shape in habitat. [file ECE3-5-5792-s004.docx]

Appendix S4. Effects of landscape structure on the evolution of dispersal propensity, path shape in matrix, and path shape in habitat.

Table S4.1. Percent sum of squares (%SS), for a multiple linear regression model of the relationship between the square-root-transformed population mean dispersal propensity after 1000 generations and the four landscape attributes, for 1000 simulation runs. We included quadratic terms for each predictor, to account for non-linear relationships. %SS combines the variance explained by both the linear and quadratic terms.

| Attribute | %SS |
| --- | --- |
| Habitat amount | 3.75 |
| Habitat fragmentation | 0.64 |
| Matrix quality | 0.50 |
| Disturbance frequency | 10.03 |
| Residual | 85.08 |

Table S4.2. Percent sum of squares (%SS), for a multiple linear regression model of the relationship between the square-root-transformed population mean path straightness in matrix after 1000 generations and the four landscape attributes, for 1000 simulation runs. We included quadratic terms for each predictor, to account for non-linear relationships. %SS combines the variance explained by both the linear and quadratic terms.

| Attribute | %SS |
| --- | --- |
| Habitat amount | 31.16 |
| Habitat fragmentation | 2.52 |
| Matrix quality | 0.59 |
| Disturbance frequency | 22.80 |
| Residual | 42.93 |

Table S4.3. Percent sum of squares (%SS), for a multiple linear regression model of the relationship between the square-root-transformed population mean path straightness in habitat after 1000 generations and the four landscape attributes, for 1000 simulation runs. We included quadratic terms for each predictor, to account for non-linear relationships. %SS combines the variance explained by both the linear and quadratic terms.

| Attribute | %SS |
| --- | --- |
| Habitat amount | 38.72 |
| Habitat fragmentation | 2.78 |
| Matrix quality | 1.31 |
| Disturbance frequency | 17.11 |
| Residual | 40.08 |





Fig. S4.1. Effects of (a) habitat amount, (b) habitat fragmentation, (c) matrix quality, and (d) disturbance frequency on the evolved dispersal propensity, when holding all other landscape attributes at their mean values. Standardized landscape attribute values were scaled such that larger values indicate more habitat, more fragmented habitat, higher matrix quality, and more frequent disturbance. Relationships were modelled by multiple linear regression, using square-root-transformed dispersal propensities (back-transformed prior to plotting), for the 1000 simulation runs.





Fig. S4.2. Effects of (a) habitat amount, (b) habitat fragmentation, (c) matrix quality, and (d) disturbance frequency on the evolved path straightness in matrix, when holding all other landscape attributes at their mean values. Standardized landscape attribute values were scaled such that larger values indicate more habitat, more fragmented habitat, higher matrix quality, and more frequent disturbance. Relationships were modelled by multiple linear regression, using square-root-transformed estimates of path straightness (back-transformed prior to plotting), for the 1000 simulation runs.





Fig. S4.3. Effects of (a) habitat amount, (b) habitat fragmentation, (c) matrix quality, and (d) disturbance frequency on the evolved path straightness in habitat, when holding all other landscape attributes at their mean values. Standardized landscape attribute values were scaled such that larger values indicate more habitat, more fragmented habitat, higher matrix quality, and more frequent disturbance. Relationships were modelled by multiple linear regression, using square-root-transformed estimates of path straightness (back-transformed prior to plotting), for the 1000 simulation runs.
