# Supplementary material for: Matrix quality and disturbance frequency drive evolution of species behavior at habitat boundaries
Source: Ecol Evol. 2015 Nov 24;5(24):5792–800. doi: 10.1002/ece3.1841 (PMC4717347; doi:10.1002/ece3.1841)
Supplement: Supplementary file 5 — Appendix S5. Example of site selection to minimize correlations between landscape attributes. [file ECE3-5-5792-s005.docx]

Appendix S5. Example of site selection to minimize correlations between landscape attributes.


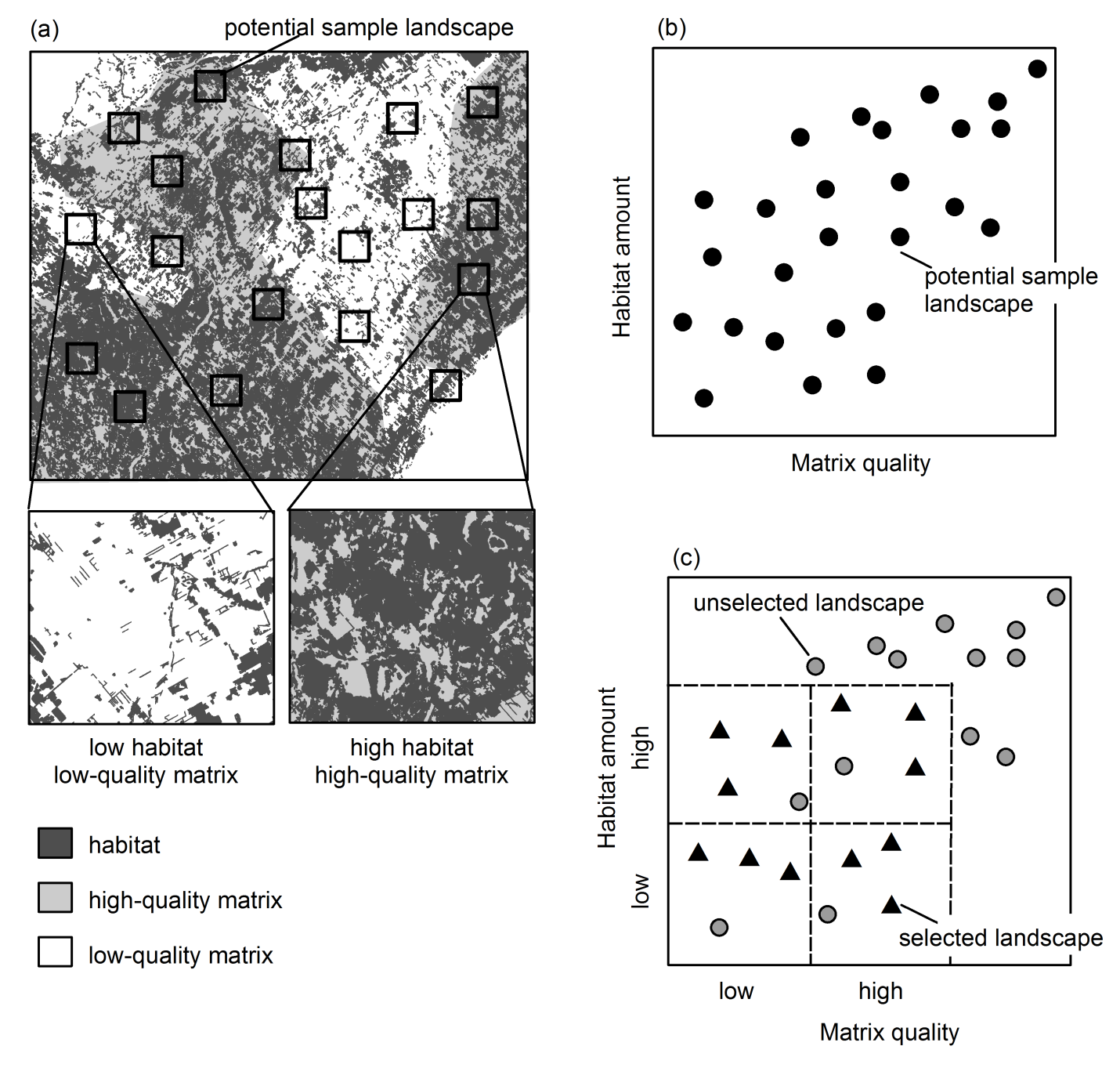


Fig. S5. Example of site selection to minimize correlations between two landscape attributes: habitat amount and matrix quality. In a given region, random selection of sample landscapes (a) results in strong, positive correlations between habitat amount and matrix quality (b). However these correlations can be minimized during site selection by defining ranges of ‘low’ and ‘high’ values of habitat amount and matrix quality, and randomly selecting an equal number of sample sites from all combinations of the low and high ranges (c).
